# A Population-Based Analysis of 30-Year Mortality among Five-Year Survivors of Adolescent and Young Adult Cancer: The Roles of Primary Cancer, Subsequent Malignancy, and Other Health Conditions

**DOI:** 10.3390/cancers13163956

**Published:** 2021-08-05

**Authors:** Diana J. Moke, Ziwei Song, Lihua Liu, Ann S. Hamilton, Dennis Deapen, David R. Freyer

**Affiliations:** 1 Cancer and Blood Disease Institute, Children’s Hospital Los Angeles, Los Angeles, CA 90027, USA; 2 Department of Pediatrics, Keck School of Medicine, University of Southern California, Los Angeles, CA 90033, USA; dfreyer@chla.usc.edu; 3 Department of Population and Public Health Sciences, Keck School of Medicine, University of Southern California, Los Angeles, CA 90033, USA; lihualiu@usc.edu (L.L.); ahamilt@med.usc.edu (A.S.H.); ddeapen@usc.edu (D.D.); 4 Los Angeles Cancer Surveillance Program, University of Southern California, Los Angeles, CA 90089, USA; ziweis@usc.edu; 5 USC Norris Comprehensive Cancer Center, University of Southern California, Los Angeles, CA 90033, USA; 6 Department of Medicine, Keck School of Medicine, University of Southern California, Los Angeles, CA 90033, USA

**Keywords:** late mortality, late effects, treatment-related toxicity, cause-specific mortality, adolescent and young adult (AYA) cancer, survivorship

## Abstract

**Simple Summary:**

Cancer survivors are at risk for developing serious health problems and dying prematurely. Adolescents and young adults (AYAs, aged 15–39 years at diagnosis) are a unique population challenged with different cancer types and treatment toxicity than other age groups, impaired access to care, financial hardship, and psychosocial distress due to their life stage. Although 85% of AYAs with cancer are alive 5 years after diagnosis, in subsequent years, their survival is consistently lower and declines faster than the general population. However, knowledge regarding why these long-term survivors of AYA cancer die prematurely is incomplete. Therefore, we sought to provide a detailed report of all causes of death in this population, including recurrence of the first cancer, development of a different cancer type, or other health problems. Our results can help inform future research to develop safer cancer treatment and better long-term care that will improve the health and survival of this vulnerable population.

**Abstract:**

Despite an aggregate 5-year survival of 85%, many adolescents and young adults (AYAs, 15–39 years old) treated for cancer die prematurely decades later. To develop a more complete understanding of this problem, particularly the role of specific subsequent malignant neoplasms (SMNs), we used the SEER-9 registry to analyze causes of death (COD: Primary cancer, SMN, non-malignant conditions) among 162,317 AYAs diagnosed with first cancer between 1975–2012 and surviving 5 or more years. Cumulative mortality, attributable mortality, standardized mortality ratios (SMRs), and adjusted hazard ratios were determined for each cancer site and COD. At 30 years, cumulative mortality due to primary cancer was matched by that due to all other causes (12.8% 95% CI [12.5%, 13.0%] for primary cancer versus 12.8% [12.5%, 13.1%] for all other causes combined) in the combined cohort, and was overtaken by non-malignant conditions in Hodgkin lymphoma, testicular, cervical/uterine, and thyroid cancers. Overall, SMNs accounted for 20% of malignant deaths, the most common being lung/bronchus (25.6%), colorectal/liver/biliary/pancreas (19.1%), and breast (10.2%). For non-malignant conditions, excess risk was noted overall (SMR 1.37, 95% CI [1.34, 1.40]) and for infectious (1.97 [1.85, 2.10]), renal (1.85 [1.60, 2.13]), cardio/cerebrovascular (1.38 [1.33, 1.43]), and suicide (1.15 [1.04, 1.27]). Racial minorities were at significantly higher risk across all COD. Safer therapy, longitudinal monitoring, and primary/secondary preventive strategies are needed to reduce late mortality in this vulnerable population.

## 1. Introduction

Almost 90,000 adolescents and young adults (AYAs, 15–39 years old) are diagnosed with cancer in the United States (US) every year [1], over eight times the number of children aged 0–14 [2]. With recent advances in cancer treatment, the survival of AYAs has steadily improved such that 85% are now living 5 years or longer following diagnosis, a higher proportion than any other age group [3,4]. Unfortunately, survival of AYA cancer patients, compared with the general population, shows persistent declines even decades later [3,5]. Because survivors of AYA cancer, like those of childhood cancer, are relatively young with the potential to live many years following treatment, an understanding of the health problems and causes of premature death in this population is required for developing effective strategies to improve their duration and quality of life.

Current knowledge regarding AYA cancer mortality, late effects, and survivorship practice is largely extrapolated from research conducted in cohorts of adult survivors of childhood cancer. Compared to the age-matched general population, survivors of childhood cancer are 8.4 times as likely to die from any cause, with elevated mortality risks due to subsequent malignant neoplasms (SMNs), as well as cardiac, pulmonary, and other medical causes [6]. For childhood cancer survivors, death from primary cancer recurrence or progression predominates during the first 10–15 years of follow-up, after which death due to late effects, including SMNs and organ toxicity, accelerates and becomes the most common cause of death by 30 years [7]. However, these observations may not be applicable to survivors of AYA cancer. Compared to children, AYAs develop cancer when co-morbidities of adulthood are emerging, have a different spectrum of malignancies, receive different treatment regimens, and exhibit distinct profiles of acute treatment-related toxicity [8,9]. Further, AYAs are more frequently burdened by multiple health disparities, including impaired access to care, suboptimal adherence to treatment, inadequate health insurance, psychosocial distress, and financial toxicity, all of which have the potential to exacerbate late effects and premature mortality [8].

Late mortality (i.e., death more than 5-years after diagnosis) after AYA cancer has begun to be addressed in recent studies, but the results are mixed and incomplete [5,10,11,12,13,14,15]. Published reports agree that early in the follow-up, recurrence of primary cancer is the leading cause of death and that recent 5-year survival improvements are the result of more effective therapy [5,15]. Beyond 5–10 years, however, the picture is less clear. Whereas studies indicate AYAs with cancer are at increased risk of dying due to non-cancer causes compared with age-matched peers [12], some have suggested this excess risk attenuates within 10 years [10]. While some studies are population-based and limited to 5-year survivors [5,10,13], others have included AYAs from diagnosis onward [11,12] or have focused on well-insured AYA cohorts with better access to care than the general population [14]. Importantly, SMNs have been suggested to impact survivors of AYA cancer disproportionately, for both incidence, as compared with childhood cancer survivors [16], and survival, as compared with older adults [17]. Despite these observations, few studies have fully examined the contribution of SMNs to all-cause mortality in AYAs, and none, to our knowledge, have delineated the types of SMNs causing death.

To address these uncertainties and limitations, we utilized data from the US National Cancer Institute’s Surveillance, Epidemiology, and End Results (SEER) Program to conduct a comprehensive study of cause-specific late mortality restricted to 5-year survivors of AYA cancer, including detailed information on the contributions of recurrent primary cancer, specific subsequent malignancies, and non-malignant causes, as well their associations with key sociodemographic factors. Our overall objective was to develop a more complete understanding of the causes of premature death among long-term survivors of AYA cancer, a young population with a long life trajectory with the potential to benefit from risk-mitigation strategies.

## 2. Materials and Methods

### 2.1. Data Source, Study Sample, and Definitions

This was a population-based study utilizing SEER-9 registry data [18]. Patients were 15–39 years old when diagnosed with their first primary cancer between 1975–2012 and survived at least 5 years as of 31 December 2016. Based on the SEER AYA Cancer Site Recode/World Health Organization 2008, the cohort was stratified by cancer site consisting of the 8 most common cancer sites among survivors from across the age spectrum of 15–39 years: Female breast, bone/soft tissue sarcoma, cervical/uterine, Hodgkin lymphoma (HL), non-Hodgkin lymphoma (NHL), melanoma, testicular germ cell tumor, thyroid, plus other, which included all other cancers [19]. Only Kaposi sarcoma was excluded because of its unique HIV-related epidemiology and mortality patterns.

Late mortality was defined as death occurring beyond 5 years from diagnosis. The underlying cause of death (i.e., Cause of Death Recode) is a SEER variable based on death certificate information coded according to the International Classification of Disease (ICD) editions 9–10 (seer.cancer.gov/codrecode/1969_d03012018/index.html, accessed on 3 August 2021). We categorized ICD-coded causes of death as follows (entities were classified under “All Other Causes” if the ICD code was “other” or the number of reported deaths was very small, typically <5–10 for the entire cohort).

- All causes (all malignant and non-malignant causes).
- Malignant (primary cancer and SMN).
- Cardio/cerebrovascular (diseases of heart, hypertension without heart disease, cerebrovascular disease, atherosclerosis, aortic aneurysm and dissection, other diseases of arteries, arterioles, capillaries).
- Infections (tuberculosis, syphilis, septicemia, other infectious and parasitic diseases including HIV, pneumonia and influenza).
- Pulmonary (chronic obstructive pulmonary disease), renal (nephritis, nephrotic syndrome, and nephrosis).
- Liver and cirrhosis (chronic liver disease and cirrhosis).
- Complications of pregnancy/childbirth (complications of pregnancy, childbirth, and puerperium).
- External causes (suicide and self-inflicted injury, accidents and adverse events, homicide and legal intervention).
- All other causes (diabetes mellitus; Alzheimer’s; stomach and duodenal ulcers; congenital anomalies; certain conditions originating in the perinatal period; symptoms, signs, and ill-defined conditions; other causes of death).

We also categorized cause of death more broadly into a study-defined variable using an additional SEER Cause-specific Death Classification (seer.cancer.gov/causespecific/, accessed on 3 August 2021) as follows:

- Dead, attributable to this cancer diagnosis (i.e., dead due to primary cancer).
- Dead, attributable to causes other than this cancer diagnosis and with a Cause of Death Recode variable of a malignancy that differed from the primary cancer diagnosis (i.e., dead due to SMN).
- Dead, attributable to causes other than this cancer diagnosis and with a Cause of Death Recode of a condition that was non-malignant (i.e., all non-malignant causes of death).

### 2.2. Statistical Methods

We constructed cause-specific mortality curves (cumulative incidence of death using Kaplan–Meier methods) conditioned on survival at 5 years from diagnosis for cases in aggregate and stratified by primary cancer site (*n* = 161,207).

We constructed multivariable Cox proportional hazards regression models and calculated adjusted hazard ratios (aHRs) with 95% confidence intervals (95% CI) by each cause of death for all AYA cancers combined to assess the impact of age at diagnosis (15–19, 20–29, or 30–39 years), sex (male or female), race (White, Black, or other (which for SEER-9 includes only Asian Pacific Islander and American Indian/Alaska Native)), and stage of disease at diagnosis (historic stage A and SEER Summary Stage: Localized, regional, distant, or unknown/unstaged). Ethnicity and socioeconomic status (SES) were unable to be included as they were not available in the SEER-9 registry dataset. In this multivariable model (total cases, *n* = 147,407), we excluded cases of unknown race (*n* = 2041) due to concerns this group could be biased and because the aHRs for other variables were virtually unchanged with or without their inclusion. We included all cancer types except for brain and central nervous system cancers (which are all unstaged, *n* = 6040), all leukemias (which are unlike solid tumors as they are all systemic, *n* = 4161), and additional cancer sites in which the stage was systematically coded as blank or unstaged in SEER-9 (nasopharynx (*n* = 577), pleura (*n* = 21), prostate (*n* = 11), mesothelioma (*n* = 44), vagina (*n* = 102), other endocrine including thymus (*n* = 391), and myeloma (*n* = 413)). For Hodgkin and non-Hodgkin lymphomas diagnosed 1983–2015, those with Ann Arbor stage I were categorized as localized, II as regional, and III or IV as distant.

We constructed attributable mortality bar graphs and compared proportions of causes of death among cancer sites using the chi-square test. We used the ICD code cause of death to calculate standardized mortality ratios (SMRs) for the observed number of deaths in this cohort compared to the expected number of deaths; this expected number was generated by the SEER analytic tool (see below) and was based on general population mortality but adjusted for the attained age, sex, and race of the site-specific cancer cohort, as well as the calendar year. We further stratified SMRs by cancer site, age, race, and time from diagnosis. Statistical operations were performed using SEER*Stat software Version 8.3.6 (Bethesda, MD, USA), SAS Version 9.4 (Cary, NC, USA), and RStudio statistical software Version 1.4.1106 (Boston, MA, USA). All tests were 2-sided with the significance set at <0.05.

## 3. Results

### 3.1. Patient Characteristics

A total of 162,317 incident cases were included in this analysis (Table 1). Survivors were followed for a median of 17.4 years (standard deviation (SD) 9.9, range 5.0–41.9). The majority of survivors were 30–39 years old at diagnosis (64.1%), female sex (62.4%), White race (83.0%), and had localized disease (58.3%). The three most common primary cancer sites were female breast (15.5%), melanoma (14.2%), and thyroid cancer (13.0%) (Table 1).

### 3.2. Cause-Specific Late Mortality

**Figure 1:**
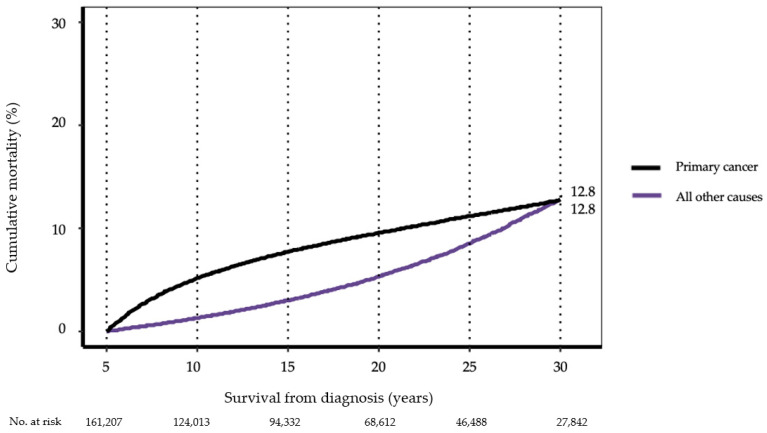
Cause-specific cumulative mortality conditioned on 5-year survival, all AYA cancers diagnosed 1975–2012, SEER-9. (See Table 2 for 95% confidence intervals at 10, 20, and 30 years.).

For the cohort as a whole, cumulative mortality from primary cancer accelerated rapidly until 10 years from diagnosis and remained the dominant cause of death until 30 years after diagnosis, when it was intersected by all other causes combined (12.8% 95% CI [12.5, 13.0%] for primary cancer versus 12.8% [12.5, 13.1%]) for all other causes combined. Mortality from all other causes combined increased steadily, especially beyond 15 years (Figure 1; Table 2).

**Figure 2:**
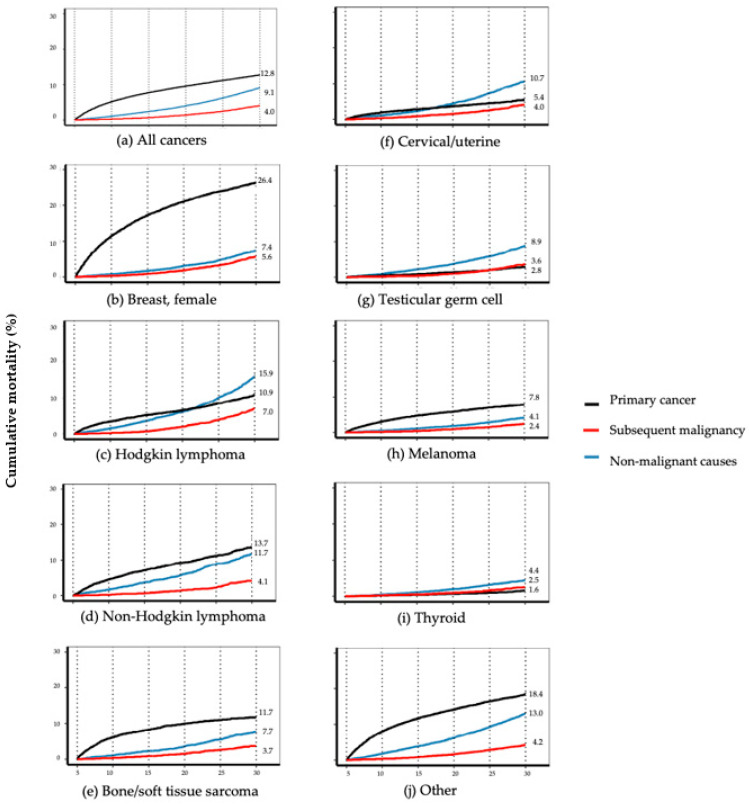

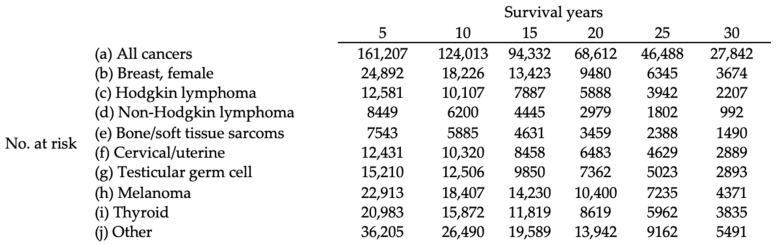
Cause-specific cumulative mortality conditioned on 5-year survival by AYA primary cancer site, diagnosed 1975–2012, SEER-9. for: (**a**) All cancers; (**b**) breast, female; (**c**) Hodgkin lymphoma; (**d**) non-Hodgkin lymphoma; (**e**) bone/soft tissue sarcoma; (**f**) cervical/uterine; (**g**) testicular germ cell; (**h**) melanoma; (**i**) thyroid; (**j**) other. (See Table 2 for 95% confidence intervals at 10, 20, and 30 years).

In differentiating all other causes of death for the overall cohort, mortality due to non-malignant causes exceeded and rose faster than SMN, beginning within 5–10 years from diagnosis and accelerating rapidly after 20 years such that it was more than double that of SMN by 30 years (9.1%, 95% CI [8.9%, 9.4%] versus 4.0% [3.9%, 4.2%]) (Figure 2a, Table 2). Cause-specific mortality trends varied substantially by cancer site. At 30 years, primary cancer was the leading cause of cumulative mortality among survivors of breast cancer (26.4% [25.7%, 27.1%]), NHL (13.7% [12.5%, 14.9%]), bone/soft tissue sarcoma (11.7% [10.9%, 12.7%]), and melanoma (7.8% [7.4%, 8.3%]) (Figure 2b,d,e,h; Table 2). In contrast, non-malignant causes led cumulative mortality at 30 years among survivors of HL (15.9% [14.9%, 17.0%]), cervical/uterine cancer (10.7% [9.9, 11.5%]), testicular germ cell (8.9% [8.2, 9.7%]), and thyroid cancer (4.4% [3.9, 4.9%]) (Figure 2c,f,g,i; Table 2). Death from causes other than primary cancer (largely non-malignant deaths) predominated by 10 years after diagnosis for testicular germ cell, by 20 years for HL and cervical/uterine cancer, and by 30 years for NHL (Figure 2c,d,f,g; Table 2). At 30 years, the mortality from SMN exceeded that of primary cancer in both testicular germ cell (3.6% [3.1%, 4.2%] versus 2.8% [2.5%, 3.3%]) and thyroid cancer (2.5% [2.1%, 2.9%] versus 1.6% [1.3%, 1.9%]) (Figure 2g,i; Table 2).

When stratified by two time periods (1975–1995 and 1996–2012), mortality from primary cancer showed a substantial reduction over time but mortality from SMNs and non-malignant causes were relatively unchanged (Figure S1).

### 3.3. Risk Factors for Late Mortality

**Figure 3:**
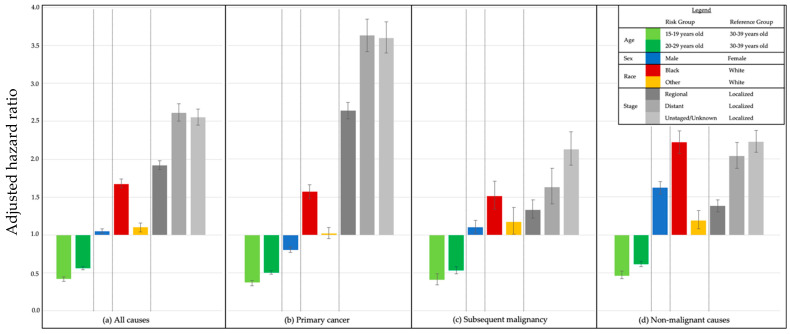
Adjusted hazard ratios by cause of death and risk group, 5-year survivors of AYA cancer diagnosed 1975–2012, SEER-9 for death from: (**a**) All causes; (**b**) primary cancer; (**c**) subsequent malignancy; and (**d**) non-malignant causes.

A total of 147,407 patients were included in the multivariable analysis of demographic risk factors impacting death due to primary cancer, SMN, and non-malignant causes (Figure 3; Table S1). Compared with patients 30–39 years old, younger age at diagnosis was significantly protective for all causes of death combined (aHR 0.56, [95% CI 0.54, 0.58] for 20–29 years old; 0.42 [0.39, 0.45] for 15–19 years old) and for primary cancer, SMN, and non-malignant causes. Males carried a significantly lower risk of death than females from primary cancer (aHR 0.80 [0.77, 0.84]) but significantly higher risks from SMN (aHR 1.10 [1.02, 1.19]) and non-malignant causes (aHR 1.62 [1.54, 1.70]). Compared to White patients, Black patients demonstrated significantly increased risks of death due to primary cancer, SMN, and non-malignant causes (aHR 1.57 [1.48, 1.66]; 1.51 [1.34, 1.71]; and 2.22 [2.07, 2.37], respectively). Compared with localized disease, both regional and distant disease carried significantly higher risks among all causes of death; these were largest for death due to primary cancer (aHR for distant disease 3.63 [3.42, 3.85]) (Figure 3 and Table S1).

### 3.4. Attributable Mortality and Risk Due to Malignant and Non-Malignant Causes

**Figure 4:**
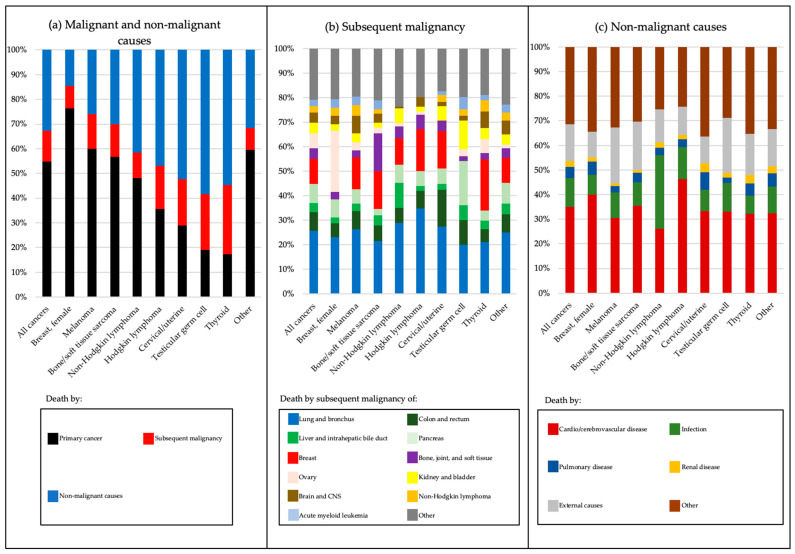
Attributable mortality among 5-year survivors of AYA cancer by cancer site and cause of death, diagnosed 1975–2012, SEER-9 for: (**a**) Death by all malignant and non-malignant causes; (**b**) death by subsequent malignancy; and (**c**) death by non-malignant causes. AYA = adolescent and young adult; CNS = central nervous system.

There were 25,999 deaths. Attributable mortality differed among survivors depending on the primary cancer type (Figure 4a–c, *p* < 0.0001 within each cause of death). Whereas the majority of deaths among survivors of breast cancer, melanoma, and bone/soft tissue sarcoma were attributable to primary cancer recurrence, survivors of thyroid cancer, testicular germ cell, cervical/uterine cancer, NHL, and HL were more likely to die of non-malignant causes or SMN (Figure 4a, Table S2A). For the overall cohort, the most common SMNs causing death were lung and bronchus cancer (25.6% combined), cancers of the digestive system (colorectal, liver and intrahepatic bile ducts, and pancreatic cancers; 19.2% combined), and breast cancer (10.2%) (Figure 4b, Table S2B). However, among breast cancer survivors, ovarian cancer was the most common SMN causing death (25.0%, Figure 4b, Table S2B). Subsequent leukemia was an uncommon cause of death by SMN across most cancer types (2.7% combined, Figure 4b, Table S2B). For non-malignant causes in the overall cohort, cardio/cerebrovascular disease was most common (34.9%), followed by external causes (suicides, accidents, homicides; 15.0% combined) and infections (11.8%) (Figure 4c, Table S2C). The proportion of non-malignant deaths attributable to cardio/cerebrovascular disease was highest among HL survivors (46.1%), whereas infectious causes were the most frequent among NHL survivors (29.9%) (Figure 4c, Table S2C).

To control for morbidities associated with aging, SMRs were determined for all causes of death (Table 3). Compared with the general population, this cohort was almost 3 times as likely to die of any cause, over 6 times as likely to die of cancer (primary cancer or SMN), and over 30% more likely to die of non-malignant causes. These risks varied considerably by the primary cancer diagnosis. While breast cancer survivors had the highest risk of death due to cancer (SMR 10.96, 95% CI [10.67, 11.26]), SMRs for all non-malignant causes combined were highest among those treated for HL (SMR 2.84 [2.68, 3.00]), NHL (SMR 2.02 [1.86, 2.19]), and cervical/uterine cancer (SMR 1.90 [1.78, 2.03]) (Table 3).

For non-malignant causes, this cohort as a whole was significantly more likely to die of every medical condition except liver/cirrhosis. The highest SMR was noted for pregnancy/childbirth (SMR 3.13, 95% CI [1.79, 5.08]), which was restricted to breast cancer survivors (SMR 4.70 [1.28, 12.02]). The risk of death by infection, nearly twice as high in the overall cohort as the general population (SMR 1.97 [1.85, 2.10]), was especially increased in survivors of NHL (SMR 7.11 [6.11, 8.23]), HL (SMR 4.18 [3.56, 4.89]), cervical/uterine cancer (SMR 2.04 [1.62, 2.54]), and testicular germ cell (SMR 1.40 [1.13, 1.72]). The risk of death by renal causes for the combined cohort (SMR 1.85 [1.60, 2.13]) was significantly increased in survivors of HL (SMR 3.65 [2.26, 5.58]), cervical/uterine cancer (SMR 2.96 [2.04, 4.16]), and NHL (SMR 2.82 [2.04, 4.16]). The risk of death by cardio/cerebrovascular causes was increased for the combined cohort as well (SMR 1.38 [1.33, 1.43]), and was especially elevated in survivors of HL (SMR 4.26 [3.91, 4.63]), cervical/uterine cancer (SMR 1.78 [1.59, 2.00]), NHL (SMR 1.54 [1.30, 1.80]), bone/soft tissue sarcoma (SMR 1.27 [1.06, 1.51]), and female breast cancer (SMR 1.22 [1.10, 1.36]). Among external causes, the risk of death by suicide was significantly increased for the cohort as a whole (SMR 1.15 [1.04, 1.27]) (Table 3).

The SMR values for non-malignant causes of death were statistically increased in all cancer sites except for testicular GCT, melanoma, and thyroid (Table 3). Three primary cancer sites stood out for having multiple, significantly increased, non-malignant, organ-related causes of death: HL, cervical/uterine cancer, and NHL (exhibiting 4, 4, and 3 such causes, respectively). The recurring causes of non-malignant death for these sites were cardio/cerebrovascular disease, infections, renal, and pulmonary causes. In contrast, both melanoma and thyroid cancers were notable for having a significantly lower risk of death for many non-malignant causes (Table 3).

Compared with older AYAs, patients diagnosed 15–19 years old had higher SMRs for almost all non-malignant causes of death compared to those aged 20–29 and 30–39 years (Table S3), except for pregnancy/childbirth complications, for which SMR was highest for older 30–39-year-olds. Patients of Black or other race also had higher SMRs for almost all causes of death compared to White patients (Table S4). SMRs for each cause of death and for each cancer type generally declined with each decade of follow-up (Table S5).

## 4. Discussion

Using the large and socio-demographically diverse SEER registry, we have presented what we believe to be the most comprehensive study to date describing causes of late mortality focused solely on 5-year survivors of AYA cancer in the US. This study encompassed not only primary cancer recurrence, SMNs in aggregate, and non-malignant causes, but also the distribution of specific SMNs, details of non-malignant causes of death by primary cancer site, and impacts of key demographics. With AYAs now experiencing higher 5-year cancer survival than any other age group, the need for a more detailed understanding of late mortality is crucial for reducing premature death through a combination of safer, more effective cancer treatment and improved survivorship care.

This study shows that for AYA survivors as a whole, the overall semblance of late mortality is time-dependent and not altogether unlike that of long-term survivors of childhood cancer [6,7]. Recurrence of primary cancer remains the most common cause of death until 30 years of follow-up, at which time it is matched, and beyond which undoubtedly exceeded, by the combination of SMNs and non-malignant causes that began to emerge 10–15 years earlier. However, this pattern varies markedly by primary cancer site. For several cancer sites, the dominant cause of death at 30 years remains recurrence, indicating a need for more effective cancer therapy. For others, including historically good-prognosis cancers such as HL and testicular germ cell, the dominant cause of death at 30 years is non-malignant conditions presumably representing late effects. In those subgroups, there is a pressing need for more research to understand how cancer therapy, comorbidities, genetics, and health behaviors conjoin as this population ages.

This study, specific to the AYA age group and focused on mortality, adds new and important information to what is known about the burden of SMNs among cancer survivors [20]. Among AYA survivors in a managed care system in the US, the most commonly diagnosed SMNs were breast cancer, melanoma, and gastrointestinal cancer [21]; whereas, in the registry-based Teenage and Young Adult Cancer Survivors Study from England and Wales, the SMN with the highest absolute excess incidence was lung cancer [22]. In contrast to those studies describing the incidence of SMNs among AYAs, this is the first study, to our knowledge, that reports attributable mortality due to SMNs by site and according to primary cancer diagnosis. Notably, the most common SMN causing death across primary sites are subsequent cancers of the lung and bronchus, which account for over one-quarter of SMN deaths in the overall cohort and over one-third in several sites. While thoracic irradiation undoubtedly accounts for substantial lung cancers in some primary sites, such as HL and breast cancer, it does not so readily explain it in others, such as cervical/uterine cancer and testicular germ cell tumor. Therefore, health behaviors beyond cancer treatment exposures require additional focused examination, such as smoke exposure and e-cigarette use, which have been reported to be elevated among survivors of AYA [23,24] but not childhood cancer [25,26]. The second most prevalent group of SMN-related deaths were those of the digestive tract (i.e., colorectal, liver/biliary, and pancreas), particularly among testicular germ cell tumors where abdominal irradiation could play a role. Breast cancer was the third most common SMN causing death in this cohort and appeared to be concentrated in primary sites where chest irradiation (e.g., HL) [27] or genetic predispositions (e.g., Li-Fraumeni syndrome with soft tissue sarcoma) could play an oncogenic role [28]. Not surprisingly, ovarian cancer, uncommon as an SMN overall, was the most common SMN causing death among breast cancer survivors, likely related to mutations of the *BRCA* genes [29]. In contrast, the relative infrequency of subsequent AML as a cause of death was somewhat unexpected but might be explained by limiting our analysis to death beyond 5 years from diagnosis.

For non-malignant causes of death, our study clarifies and extends somewhat conflicting recent literature. We found that in addition to cardio/cerebrovascular and pulmonary causes of death commonly observed in survivors of childhood cancer [6,7], AYAs exhibit substantial risk for infectious and renal causes. Our results are consistent with those of Anderson and colleagues, who described non-malignant causes of death in an AYA cancer cohort beginning at diagnosis and found increased risks for infectious, renal, and cardiovascular death that were highest shortly after diagnosis, declined somewhat thereafter, but persisted for over 20 years [12]. However, using conditional relative survival methods, the same authors also found an attenuation of excess mortality once survivors reached 7 years of follow-up [10]. We also detected reductions in SMRs over time and with increasing age at cancer diagnosis, but our cumulative mortality curves clarify this reflects increasing baseline mortality of the aging non-cancer population rather than reduced rates of death from non-malignant causes among survivors. Our findings are largely in agreement with those of a recent retrospective study by Armenian and colleagues, which examined 2-year survivors of AYA cancer within the Kaiser Permanente managed care system in Southern California. They found an elevated risk of all-cause mortality compared with matched non-cancer members, and that, starting at 15 years, survivors experienced a higher incidence of mortality from SMN or other health-related conditions that exceeded that due to primary cancer recurrence [14]. That study differed from ours by not restricting their population to 5-year survivors, having a shorter duration of follow-up (starting at 1992 rather than 1980), and drawing its cohort from a private, insurance-based system, which may limit generalizability to AYA populations burdened by less favorable insurance and reduced access to appropriate care. Using population-based data, we were able to validate the elevated risks of malignant and non-malignant death among AYA survivors, while also addressing generalizability concerns, which is important because AYAs constitute a cancer population singularly burdened with health care disparities [30].

Regarding sociodemographic factors, our study found that survivors of AYA cancer who were diagnosed at older ages, were of Black race, and had certain primary cancer sites were at significantly higher risk for premature death. These findings are consistent with our previous study of AYAs from the California Cancer Registry showing significantly lower 5-year survival for Hispanic, Black, and low SES patients [3]. They are also similar to those of recent studies using data from SEER [11] and the Texas Cancer Registry [30], which demonstrate adverse impacts on 5-year survival for patients who are non-Hispanic Black or Hispanic race/ethnicity, impoverished, or under-insured. The current study highlights the perniciousness of known racial disparities long after treatment and underscores the urgency of identifying underlying societal health disparities and understanding barriers particularly impactful for AYAs with cancer.

This study has several strengths and some limitations. Strengths include the use of the SEER-9 database, a large, high-quality, and widely used cancer registry that is highly representative of the US population with decades of follow-up since the 1970s, features that maximize external validity and generalizability of our conclusions. Determining the specific SMNs causing death is, to our knowledge, novel. Additionally, by restricting our analysis to 5-year survivors with over three decades of follow-up, our study provides results that are highly relevant to this young population as it moves through successive life stages. Most of the limitations are inherent to registry-based studies, principally risk for misclassification. However, the cause of death by ICD code is unlikely to be biased as it is documented in the physician-certified death certificate. Furthermore, SEER has defined the Cause-specific Death Classification, an improved and validated algorithm recode that accounts for cause of death in conjunction with the primary cancer site, the sequence of multiple tumors (in a setting where over 90% of sequential cancers are microscopically confirmed), and diseases related to the cancer of diagnosis [20,31,32]. By including patients diagnosed as far back as 1975 we maximized the duration of follow-up, which permitted an accurate and complete accounting of causes of late mortality, but we were unable to examine the effects of ethnicity and socioeconomic status because these were not included as SEER variables prior to 2000. Similarly, SEER does not collect data regarding insurance status over time or other barriers to care. Additionally, because SEER captures only limited data on initial cancer treatment, we were unable to determine associations of treatment with causes of death. Finally, we have not comprehensively examined mortality trends over time, which may be useful for assessing the impacts of the treatment era but are beyond the scope of this analysis.

## 5. Conclusions

This study synthesizes, clarifies, and extends current knowledge about the causes of late mortality among long-term survivors of AYA cancer. As these results make clear, the only generalization that holds for 5-year survivors is that, across all AYA sites except thyroid, recurrence of the primary cancer is the most common cause of death during the first decade. This indicates that control of primary cancer through improved diagnosis and treatment remains a high priority for many AYA cancers. Beyond 10–15 years, our study shows that late mortality patterns differ markedly by primary cancer. For some types, recurrence remains the chief hazard, whereas for others it is evident that SMNs and late toxicity are major causes. These observations should encourage a more nuanced, cancer-specific approach to determining which host- and treatment-related risk factors contribute most to late mortality. Finally, the findings of this study, which document the unrelenting burden of late mortality over time from all causes, pose an urgent and compelling argument for expanding the availability of systematic, risk-adapted, longitudinal follow-up of AYA survivors [33,34]. Developing these services can draw on decades of successful experience with long-term survivors of childhood cancer [35,36,37], although differences in the types, frequencies, and mechanisms of late effects in AYAs, along with understanding the disparities and barriers to health care faced specifically by AYAs, will require the development of more specific monitoring guidelines and care models appropriate to this population.

## Figures and Tables

**Table 1:** Demographics and tumor characteristics, 5-year survivors of AYA cancer diagnosed 1975–2012, SEER-9.

Characteristic	*n* (%) or (Standard Deviation)
Total	162,317
Median follow-up, years	17.4 (9.9)
Age, years	
15–19	10,493 (6.5)
20–29	47,760 (29.4)
30–39	104,064 (64.1)
Sex	
Male	61,085 (37.6)
Female	101,232 (62.4)
Race	
White	134,816 (83.0)
Black	13,388 (8.2)
API/AI/AN	12,054 (7.4)
Unknown	2059 (1.3)
Cancer site	
Bone/soft tissue sarcoma ^1^	7589 (4.7)
Breast, female	25,104 (15.5)
Cervical/uterine	12,538 (7.7)
Hodgkin lymphoma	12,687 (7.8)
Non-Hodgkin lymphoma	8502 (5.2)
Melanoma	23,039 (14.2)
Testicular germ cell	15,274 (9.4)
Thyroid	21,055 (13.0)
Other ^1^	36,529 (22.5)
Stage	
Localized	94,699 (58.3)
Regional	37,639 (23.2)
Distant	16,099 (9.9)
Unstaged/Unknown	13,869 (8.5)
Not recorded	11 (0.0)

^1^ Excluding Kaposi sarcoma; AYA = adolescent and young adult; API = Asian/Pacific Islander; AI = American Indian; AN = Alaska Native.

**Table 2:** Point estimates of 10, 20, and 30-year mortality by AYA cancer site and cause of death, diagnosed 1975–2012, SEER-9.

Primary Cancer Site	*n*	10-Year Cumulative Mortality % [95% CI]	20-Year Cumulative Mortality % [95% CI]	30-Year Cumulative Mortality % [95% CI]
Death due to primary cancer				
All cancers ^1^	161,207 ^2^	5.2 [5.0, 5.3]	9.6 [9.4, 9.7]	12.8 [12.5, 13.0]
Breast, female	24,892	11.5 [11.1, 11.9]	21.0 [20.4, 21.6]	26.4 [25.7, 27.1]
Hodgkin lymphoma	12,581	3.4 [3.1, 3.7]	6.6 [6.1, 7.1]	10.9 [10.1, 11.7]
Non-Hodgkin lymphoma	8449	4.5 [4.0, 5.0]	9.2 [8.5, 10.0]	13.7 [12.5, 14.9]
Bone/soft tissue sarcoma ^1^	7543	6.1 [5.6, 6.7]	10.0 [9.2, 10.7]	11.7 [10.9, 12.7]
Cervical/uterine	12,431	1.9 [1.6, 2.1]	3.6 [3.3, 4.0]	5.4 [4.9, 6.0]
Testicular germ cell	15,210	0.4 [0.3, 0.5]	1.4 [1.2, 1.6]	2.8 [2.5, 3.3]
Melanoma	22,913	3.0 [2.8, 3.2]	5.8 [5.4, 6.1]	7.8 [7.4, 8.3]
Thyroid	20,983	0.2 [0.1, 0.3]	0.7 [0.6, 0.9]	1.6 [1.3, 1.9]
Other ^1^	36,205	8.0 [7.7, 8.3]	14.1 [13.7, 14.6]	18.4 [17.8, 18.9]
Death due to subsequent malignancy				
All cancers ^1^	161,207 ^2^	0.2 [0.2, 0.3]	1.4 [1.3, 1.5]	4.0 [3.9, 4.2]
Breast, female	24,892	0.3 [0.2, 0.4]	1.8 [1.6, 2.0]	5.6 [5.1, 6.1]
Hodgkin lymphoma	12,581	0.2 [0.2, 0.3]	2.0 [1.7, 2.3]	7.0 [6.3, 7.8]
Non-Hodgkin lymphoma	8449	0.2 [0.1, 0.4]	1.3 [1.0, 1.7]	4.1 [3.3, 5.1]
Bone/soft tissue sarcoma ^1^	7543	0.4 [0.3, 0.6]	1.5 [1.2, 1.9]	3.7 [3.1, 4.5]
Cervical/uterine	12,431	0.3 [0.2, 0.5]	1.5 [1.3, 1.8]	4.0 [3.5, 4.6]
Testicular germ cell	15,210	0.1 [0.1, 0.2]	1.0 [0.8, 1.2]	3.6 [3.1, 4.2]
Melanoma	22,913	0.1 [0.1, 0.2]	0.9 [0.7, 1.0]	2.4 [2.1, 2.7]
Thyroid	20,983	0.2 [0.2, 0.3]	0.9 [0.8, 1.1]	2.5 [2.1, 2.9]
Other ^1^	36,205	0.3 [0.3, 0.4]	1.6 [1.4, 1.8]	4.2 [3.8, 4.6]
Death due to non-malignant causes				
All cancers ^1^	161,207 ^2^	1.1 [1.0, 1.1]	4.0 [3.9, 4.1]	9.1 [8.9, 9.4]
Breast, female	24,892	0.8 [0.6, 0.9]	3.0 [2.8, 3.3]	7.4 [6.8, 8.0]
Hodgkin lymphoma	12,581	1.5 [1.3, 1.8]	6.1 [5.6, 6.6]	15.9 [14.9, 17.0]
Non-Hodgkin lymphoma	8449	1.6 [1.4, 2.0]	5.7 [5.1, 6.4]	11.7 [10.5, 13.0]
Bone/soft tissue sarcoma ^1^	7543	1.1 [0.9, 1.4]	3.6 [3.1, 4.1]	7.7 [6.8, 8.6]
Cervical/uterine	12,431	1.0 [0.8, 1.2]	4.3 [3.9, 4.8]	10.7 [9.9, 11.5]
Testicular germ cell	15,210	0.9 [0.7, 1.0]	3.7 [3.4, 4.1]	8.9 [8.2, 9.7]
Melanoma	22,913	0.5 [0.4, 0.6]	1.8 [1.6, 2.0]	4.1 [3.7, 4.5]
Thyroid	20,983	0.5 [0.4, 0.6]	1.9 [1.7, 2.2]	4.4 [3.9, 4.9]
Other ^1^	36,205	1.8 [1.6, 1.9]	6.2 [5.9, 6.5]	13.0 [12.5, 13.7]

^1^ Excluding Kaposi sarcoma; ^2^ Not included in cumulative mortality analysis = 1100 (1099 with cause of death not available, and 11 prostate cases with stage not recorded); AYA = adolescent and young adult; 95% CI = 95% confidence interval.

**Table 3:** Standardized mortality ratios for death after 5 years for AYA cancer survivors by cancer site and ICD coded cause of death, diagnosed 1975–2012, SEER-9.

Primary Cancer Site	Data Shown in Cause of Death Columns	All Causes	Malignant Causes	All Non- Malignant Causes	Cardio/Cerebrovascular	Infections	Pulmonary	Renal	Liver and Cirrhosis	Pregnancy and Childbirth	Suicide	Accidents	Homicides and Legal Intervention	All Other Coded Causes
All cancers ^1^	Obs	25,999	17,280	8378	2928	986	379	196	277	16	380	800	80	2336
	SMR [95% CI]	**2.93** **[2.90, 2.97]**	**6.35** **[6.26, 6.45]**	**1.37** **[1.34, 1.40]**	**1.38** **[1.33, 1.43]**	**1.97** **[1.85, 2.10]**	**1.11** **[1.00, 1.23]**	**1.85** **[1.60, 2.13]**	0.94 [0.83, 1.06]	**3.13** **[1.79, 5.08]**	**1.15** **[1.04, 1.27]**	1.02 [0.95, 1.09]	**0.71** **[0.56, 0.88]**	**1.55** **[1.49, 1.62]**
Breast, female	Obs	6357	5426	917	366	75	49	15	26	4	27	62	8	285
	SMR [95% CI]	**4.76** **[4.65, 4.88]**	**10.96** **[10.67, 11.26]**	**1.10** **[1.03, 1.17]**	**1.22** **[1.10, 1.36]**	1.13 [0.89, 1.41]	**0.74** **[0.55, 0.98]**	0.78 [0.44, 1.28]	0.76 [0.50, 1.11]	**4.70** **[1.28, 12.02]**	1.09 [0.72, 1.59]	0.85 [0.65, 1.09]	0.93 [0.40, 1.83]	**1.19** **[1.05, 1.33]**
Hodgkin lymphoma	Obs	2583	1368	1206	557	159	38	21	19	1	41	93	5	272
	SMR [95% CI]	**4.50** **[4.33, 4.68]**	**9.35** **[8.87, 9.86]**	**2.84** **[2.68, 3.00]**	**4.26** **[3.91, 4.63]**	**4.18** **[3.56, 4.89]**	**2.43** **[1.72, 3.33]**	**3.65** **[2.26, 5.58]**	0.90 [0.54, 1.41]	2.01 [0.05, 11.18]	1.30 [0.93, 1.76]	**1.27** **[1.02, 1.55]**	**0.38** **[0.12, 0.89]**	**2.86** **[2.53, 3.22]**
Non-Hodgkin lymphoma	Obs	1454	846	602	157	180	19	13	13	0	23	50	8	139
	SMR [95% CI]	**3.55** **[3.37, 3.73]**	**7.66** **[7.15, 8.20]**	**2.02** **[1.86, 2.19]**	**1.54** **[1.30, 1.80]**	**7.11** **[6.11, 8.23]**	1.46 [0.88, 2.28]	**2.82** **[1.50, 4.83]**	0.87 [0.46, 1.49]	0.00 [0.00, 17.80]	1.22 [0.78, 1.84]	1.15 [0.85, 1.51]	1.06 [0.46, 2.09]	**2.05** **[1.73, 2.42]**
Bone/soft tissue sarcoma ^1^	Obs	1256	866	373	132	36	14	4	12	2	19	48	7	99
	SMR [95% CI]	**2.97** **[2.81, 3.14]**	**7.37** **[6.89, 7.88]**	**1.23** **[1.11, 1.36]**	**1.27** **[1.06, 1.51]**	1.31 [0.92, 1.82]	1.00 [0.55, 1.67]	0.77 [0.21, 1.97]	0.86 [0.44, 1.50]	7.40 [0.90, 26.72]	1.09 [0.66, 1.71]	1.13 [0.84, 1.50]	0.84 [0.34, 1.73]	**1.40** **[1.14, 1.70]**
Cervical/ uterine	Obs	1774	844	923	307	80	67	33	23	1	20	73	8	311
	SMR [95% CI]	**2.29** **[2.19, 2.40]**	**2.96** **[2.77, 3.17]**	**1.90** **[1.78, 2.03]**	**1.78** **[1.59, 2.00]**	**2.04** **[1.62, 2.54]**	**1.80** **[1.39, 2.28]**	**2.96** **[2.04, 4.16]**	1.15 [0.73, 1.73]	1.70 [0.04, 9.50]	1.31 [0.80, 2.03]	**1.64** **[1.29, 2.07]**	1.40 [0.61, 2.76]	**2.23** **[1.99, 2.49]**
Testicular germ cell	Obs	1384	571	801	265	93	18	15	48	-	70	98	11	183
	SMR [95% CI]	**1.31** **[1.24, 1.38]**	**2.29** **[1.20, 2.48]**	1.00 [0.93, 1.07]	0.96 [0.85, 1.09]	**1.40** **[1.13, 1.72]**	**0.62** **[0.37, 0.98]**	1.51 [0.85, 2.49]	1.07 [0.79, 1.42]	-	1.11 [0.87, 1.41]	**0.76** **[0.61, 0.92]**	0.64 [0.32, 1.14]	1.10 [0.95, 1.27]
Melanoma	Obs	2134	1568	553	169	57	15	5	18	3	55	65	6	160
	SMR [95% CI]	**1.57** **[1.50, 1.64]**	**3.69** **[3.51, 3.88]**	**0.60** **[0.55, 0.65]**	**0.52** **[0.44, 0.60]**	0.82 [0.62, 1.06]	**0.27** **[0.15, 0.44]**	**0.34** **[0.11, 0.80]**	**0.38** **[0.22, 0.60]**	4.50 [0.93, 13.15]	1.04 [0.78, 1.35]	**0.55** **[0.42, 0.70]**	**0.46** **[0.17, 0.99]**	**0.70** **[0.60, 0.82]**
Thyroid	Obs	945	427	514	166	37	26	18	18	1	26	58	2	162
	SMR [95% CI]	0.96 [0.90, 1.03]	**1.33** **[1.21, 1.46]**	**0.78** **[0.72, 0.85]**	**0.75** **[0.64, 0.87]**	**0.72** **[0.51, 0.99]**	**0.65** **[0.42, 0.95]**	1.53 [0.90, 2.41]	**0.57** **[0.34, 0.90]**	1.03 [0.03, 5.76]	0.78 [0.51, 1.15]	**0.70** **[0.53, 0.90]**	**0.19** **[0.02, 0.68]**	0.95 [0.81, 1.11]
Other ^1^	Obs	8112	5364	2489	809	269	133	72	100	4	99	253	25	725
	SMR [95% CI]	**4.16** **[4.07, 4.26]**	**9.40** **[9.15, 9.66]**	**1.82** **[1.75, 1.89]**	**1.65** **[1.54, 1.77]**	**2.33** **[2.06, 2.62]**	**1.90** **[1.59, 2.26]**	**3.04** **[2.38, 3.83]**	**1.52** **[1.24, 1.85]**	**3.76** **[1.02, 9.62]**	**1.35** **[1.09, 1.64]**	**1.44** **[1.27, 1.63]**	0.88 [0.57, 1.30]	**2.23** **[2.07, 2.40]**

^1^ Excluding Kaposi sarcoma. AYA = adolescent and young adult; Obs = observed deaths; SMR = standardized mortality ratio; 95% CI = 95% confidence interval. Note: Bolded SMRs are statistically significant.

## Data Availability

The data presented in this study are open and available in the SEER-9 registry, reference [18].

## Supplementary Materials

The following are available online at https://www.mdpi.com/article/10.3390/cancers13163956/s1, Figure S1: Cause-specific mortality by cause of death and time period conditioned on 5-year survival, all AYA cancers diagnosed 1975–2012, SEER-9; Table S1: Multivariable analysis of patient and tumor factors for death by cause of death, 5-year survivors of AYA cancer diagnosed 1975–2012, SEER-9; Table S2: Attributable mortality by cause of death, 5-year survivors of AYA cancer diagnosed 1975–2012, SEER-9; Table S3: Observed numbers and standardized mortality ratios by cause of death and age group at diagnosis, 5-year survivors of all AYA cancers diagnosed 1975–2012, SEER-9; Table S4: Observed cases and standardized mortality ratios by cause of death and race, 5-year survivors of all AYA cancers diagnosed 1975–2012, SEER-9; Table S5: Observed numbers and standardized mortality ratios by cause of death and latency from diagnosis through 30 years, 5-year survivors of all AYA cancers diagnosed 1975–2012, SEER-9.
