# Supplementary material for: A Population-Based Analysis of 30-Year Mortality among Five-Year Survivors of Adolescent and Young Adult Cancer: The Roles of Primary Cancer, Subsequent Malignancy, and Other Health Conditions
Source: Cancers (Basel). 2021 Aug 5;13(16):3956. doi: 10.3390/cancers13163956 (PMC8394271; doi:10.3390/cancers13163956)
Supplement: Supplementary file 1 [file cancers-13-03956-s001.zip › cancers-1288122-supplementary.pdf]

Table S1. Multivariable analysis of patient and tumor factors for death by cause of death, 5-year survivors of AYA cancer diagnosed 1975–2012, SEER-9

		n	aHR [95%CI]	*p*-value
	Cause of Death: All			
Age (years)	30–39	96,264	Ref	–
	20–29	42,791	0.56 [0.54, 0.58]	<0.0001
	15–19	8352	0.42 [0.39, 0.45]	<0.0001
Sex	Female	94,242	Ref	–
	Male	53,165	1.05 [1.02, 1.08]	0.0009
Race	White	124,481	Ref	–
	Black	12,188	1.68 [1.62, 1.75]	<0.0001
	Other[1]	10,738	1.10 [1.04, 1.16]	0.0006
Stage	Localized	89,763	Ref	–
	Regional	36,303	1.92 [1.86, 1.98]	<0.0001
	Distant	11,064	2.61 [2.50, 2.73]	<0.0001
	Unstaged/unknown	10,277	2.55 [2.45, 2.66]	<0.0001
	Cause of death: Primary cancer			
Age	30–39 years	88,854	Ref	–
	20–29 years	40,190	0.50 [0.48, 0.53]	<0.0001
	15–19 years	7895	0.37 [0.33, 0.40]	<0.0001
Sex	Female	88,387	Ref	–
	Male	48,552	0.80 [0.77, 0.84]	<0.0001
Race	White	115,941	Ref	–
	Black	10,893	1.57 [1.48, 1.66]	<0.0001
	Other[1]	10,105	1.02 [0.95, 1.10]	0.59
Stage	Localized	84,286	Ref	–
	Regional	33,931	2.64 [2.53, 2.75]	<0.0001
	Distant	10,159	3.63 [3.42, 3.85]	<0.0001
	Unstaged/unknown	8563	3.60 [3.40, 3.81]	<0.0001
	Cause of death: Subsequent malignancy			
Age	30–39 years	82,119	Ref	–
	20–29 years	38,531	0.53 [0.49, 0.58]	<0.0001
	15–19 years	7618	0.41 [0.34, 0.49]	<0.0001
Sex	Female	82,165	Ref	–
	Male	46,103	1.10 [1.02, 1.19]	0.009
Race	White	108,982	Ref	–
	Black	9770	1.51 [1.34, 1.71]	<0.0001
	Other[1]	9516	1.17 [1.01, 1.36]	0.031
Stage	Localized	81,542	Ref	–
	Regional	30,444	1.33 [1.22, 1.46]	<0.0001
	Distant	8929	1.63 [1.41, 1.88]	<0.0001
	Unstaged/unknown	7353	2.13 [1.92, 2.36]	<0.0001
	Cause of death: Non-malignant causes			
Age	30–39 years	84,945	Ref	–
	20–29 years	39,694	0.61 [0.58, 0.65]	<0.0001
	15–19 years	7825	0.46 [0.42, 0.52]	<0.0001
Sex	Female	84,084	Ref	–
	Male	48,380	1.62 [1.54, 1.70]	<0.0001
Race	White	112,242	Ref	–
	Black	10,467	2.22 [2.07, 2.37]	<0.0001

	Other[1]	9755	1.19 [1.08, 1.32]	0.0004
Stage	Localized	83,579	Ref	–
	Regional	31,402	1.38 [1.30, 1.46]	<0.0001
	Distant	9404	2.04 [1.88, 2.22]	<0.0001
	Unstaged/unknown	8079	2.23 [2.09, 2.38]	<0.0001

[1] Other race = Asian/Pacific Islander/American Indian/Alaska Native. AYA = adolescent and young adult. aHR = adjusted hazard ratio; 95%CI = 95% confidence interval; Ref = reference group.

Table S2. Attributable mortality by cause of death, 5-year survivors of AYA cancer diagnosed 1975–2012, SEER-9

A. Death from all causes

Primary cancer site	All causes of death	Primary cancer	Subsequent malignancy	Non-malignant causes
All Cancers	25,658	14,052 (54.8)	3228 (12.6)	8378 (32.6)
Breast, female	6343	4843 (76.4)	583 (9.2)	917 (14.4)
Melanoma	2121	1272 (60.0)	296 (14.0)	553 (26.1)
Bone/soft tissue sarcoma	1239	704 (56.8)	162 (13.1)	373 (30.1)
Non-Hodgkin lymphoma	1448	698 (48.2)	148 (10.2)	602 (41.6)
Hodgkin lymphoma	2574	918 (35.7)	450 (17.5)	1206 (46.8)
Cervical/uterine	1767	512 (29.0)	332 (18.8)	923 (52.2)
Testicular germ cell	1372	261 (19.0)	310 (22.6)	801 (58.4)
Thyroid	941	162 (17.2)	265 (28.2)	514 (54.6)
Other	7853	4682 (59.6)	682 (8.7)	2489 (31.7)

B. Death by subsequent malignancy

Primary cancer site	All subsequent malignancies	Lung and bronchus	Colon and rectum	Liver and intrahepatic bile duct	Pancreas	Breast	Bone, joint, and soft tissue	Ovary	Kidney and bladder	Brain and CNS	Non-Hodgkin lymphoma	Acute myeloid leukemia	Other
All Cancers	3228	828 (25.6)	250 (7.7)	120 (3.7)	249 (7.7)	331 (10.2)	139 (4.3)	194 (6.1)	140 (4.3)	136 (4.2)	85 (2.6)	86 (2.7)	670 (20.8)
Breast, female	583	135 (23.2)	32 (5.5)	14 (2.4)	44 (7.5)	0 (0.0)	17 (2.9)	146 (25.0)	15 (2.6)	21 (3.6)	18 (3.1)	21 (3.6)	120 (20.6)
Melanoma	296	78 (26.4)	22 (7.4)	9 (3.0)	17 (5.7)	39 (13.2)	8 (2.7)	10 (3.4)	11 (3.7)	21 (7.1)	13 (4.4)	10 (3.4)	58 (19.6)
Bone/soft tissue sarcoma	162	35 (21.6)	10 (6.2)	7 (4.3)	4 (2.5)	25 (15.4)	25 (15.4)	4 (2.5)	3 (1.8)	6 (3.7)	3 (1.8)	6 (3.7)	34 (21.0)
Non-Hodgkin lymphoma	148	43 (29.0)	9 (6.1)	15 (10.1)	11 (7.4)	16 (10.8)	7 (4.7)	2 (1.4)	9 (6.1)	1 (0.7)	0 (0.0)	0 (0.0)	35 (23.6)
Hodgkin lymphoma	450	157 (34.9)	32 (7.1)	9 (2.0)	27 (6.0)	77 (17.1)	27 (6.0)	6 (1.3)	8 (1.8)	18 (4.0)	0 (0.0)	0 (0.0)	89 (19.8)
Cervical/uterine	332	91 (27.4)	50 (15.1)	8 (2.4)	21 (6.3)	50 (15.1)	15 (4.5)	0 (0.0)	19 (5.7)	6 (1.8)	9 (2.7)	5 (1.5)	58 (17.5)
Testicular germ cell	310	62 (20.0)	31 (10.0)	19 (6.1)	56 (18.1)	0 (0.0)	6 (1.9)	9 (2.9)	36 (11.6)	6 (1.9)	8 (2.6)	16 (5.2)	61 (19.7)
Thyroid	265	56 (21.1)	14 (5.3)	9 (3.4)	11 (4.2)	55 (20.8)	7 (2.6)	15 (5.7)	12 (4.5)	18 (6.8)	12 (4.5)	6 (2.3)	50 (18.9)
Other	682	171 (25.1)	50 (7.3)	30 (4.4)	58 (8.5)	69 (10.1)	27 (4.0)	11 (1.6)	27 (4.0)	39 (5.7)	22 (3.2)	22 (3.2)	156 (22.9)

C. Death by non-malignant causes

**Primary cancer site**	**All non-malignant causes**	**Cardio/cerebrovascular disease**	**Infection**	**Pulmonary disease**	**Renal disease**	**External causes**	**Other**
All Cancers	8378	2928 (34.9)	986 (11.8)	379 (4.5)	196 (2.3)	1260 (15.0)	2629 (31.4)
Breast, female	917	366 (39.9)	75 (8.2)	49 (5.3)	15 (1.6)	97 (10.6)	315 (34.4)
Melanoma	553	169 (30.6)	57 (10.3)	15 (2.7)	5 (0.9)	126 (22.8)	181 (32.7)
Bone/soft tissue sarcoma	373	132 (35.4)	36 (9.6)	14 (3.8)	4 (1.1)	74 (19.8)	113 (30.3)
Non-Hodgkin lymphoma	602	157 (26.1)	180 (29.9)	19 (3.2)	13 (2.2)	81 (13.4)	152 (25.2)
Hodgkin lymphoma	1206	557 (46.2)	159 (13.2)	38 (3.2)	21 (1.7)	139 (11.5)	292 (24.2)
Cervical/uterine	923	307 (33.3)	80 (8.7)	67 (7.2)	33 (3.6)	101 (10.9)	335 (36.3)
Testicular germ cell	801	265 (33.1)	93 (11.6)	18 (2.2)	15 (1.9)	179 (22.3)	231 (28.8)
Thyroid	514	166 (32.3)	37 (7.2)	26 (5.0)	18 (3.5)	86 (16.7)	181 (35.2)
Other	2489	809 (32.5)	269 (10.8)	133 (5.3)	72 (2.9)	377 (15.1)	829 (33.3)

AYA = adolescent and young adult.

Table S3. Observed numbers and standardized mortality ratios by cause of death and age group at diagnosis, 5-year survivors of all AYA cancers diagnosed 1975–2012, SEER-9

Cause of death	15–19 years		20–24 years		25–29 years		30–34 years		35–39 years	
	Obs	SMR [95%CI]	Obs	SMR [95%CI]	Obs	SMR [95%CI]	Obs	SMR [95%CI]	Obs	SMR [95%CI]
All causes of death	1146	**4.59 [4.33, 4.86]**	2144	**3.71 [3.56, 3.87]**	4027	**3.18 [3.08, 3.28]**	6898	**2.87 [2.80, 2.94]**	11,784	**2.70 [2.65, 2.75]**
Malignant cancers[1]	704	**16.94 [15.71, 18.24]**	1269	**10.09 [9.54, 10.66]**	2570	**7.50 [7.21, 7.80]**	4698	**6.29 [6.11, 6.48]**	8039	**5.49 [5.37, 5.61]**
Cardio/cerebrovascular	126	**3.30 [2.75, 3.93]**	270	**2.45 [2.17, 2.77]**	457	**1.65 [1.50, 1.81]**	715	**1.23 [1.15, 1.33]**	1360	**1.22 [1.15, 1.28]**
Infections	56	**3.32 [2.51, 4.31]**	99	**2.36 [1.91, 2.87]**	194	**2.31 [2.00, 2.66]**	271	**1.96 [1.74, 2.21]**	366	**1.67 [1.50, 1.85]**
Pulmonary	10	**2.96 [1.42, 5.44]**	16	1.42 [0.81, 2.30]	46	1.31 [0.96, 1.74]	92	1.03 [0.83, 1.26]	215	1.06 [0.93, 1.21]
Renal	10	**5.51 [2.64, 10.13]**	14	**2.80 [1.53, 4.70]**	33	**2.55 [1.76, 3.58]**	43	**1.51 [1.09, 2.04]**	96	**1.66 [1.35, 2.03]**
Liver and cirrhosis	11	1.50 [0.75, 2.69]	27	1.27 [0.84, 1.85]	47	0.98 [0.72, 1.30]	81	0.96 [0.76, 1.19]	111	**0.83 [0.69, 1.00]**
Complications of pregnancy/childbirth	1	1.58 [0.04, 8.82]	3	3.15 [0.65, 9.20]	2	1.58 [0.19, 5.70]	5	**4.03 [1.31, 9.39]**	5	**4.91 [1.59, 11.46]**
Suicide and self-inflicted Injury	29	1.21 [0.81, 1.74]	55	1.30 [0.98, 1.69]	65	0.98 [0.75, 1.24]	100	1.14 [0.93, 1.38]	131	**1.20 [1.00, 1.42]**
Accidents and adverse events	60	1.00 [0.76, 1.29]	123	**1.23 [1.02, 1.47]**	149	0.97 [0.82, 1.14]	196	0.96 [0.83, 1.10]	272	1.03 [0.91, 1.16]
Homicide and legal intervention	9	0.65 [0.30, 1.24]	12	0.65 [0.33, 1.13]	18	0.75 [0.45, 1.19]	15	0.56 [0.31, 0.92]	26	0.88 [0.57, 1.29]
All other causes	95	**2.30 [1.86, 2.81]**	218	**2.23 [1.94, 2.54]**	364	**1.68 [1.51, 1.86]**	607	**1.48 [1.37, 1.61]**	1052	**1.42 [1.34, 1.51]**

[1] Includes primary cancer and subsequent malignancy. AYA = adolescent and young adult. SMR = standardized mortality ratio; Obs = observed number of deaths. 95%CI = 95% confidence interval.

Table S4. Observed cases and standardized mortality ratios by cause of death and race, 5-year survivors of all AYA cancers diagnosed 1975–2012, SEER-9

Cause of death	White		Black		Other**	
	Obs	SMR [95%CI]	Obs	SMR [95%CI]	Obs	SMR [95%CI]
All causes of death	21,190	**2.79 [2.75, 2.83]**	3089	**3.18 [3.07, 3.29]**	1720	**5.92 [5.65, 6.21]**
Malignant cancers[1]	14,179	**5.98 [5.89, 6.08]**	1909	**7.57 [7.23, 7.92]**	1192	**12.12 [11.44, 12.83]**
Cardio/cerebrovascular	2298	**1.30 [1.25, 1.35]**	448	**1.57 [1.43, 1.72]**	182	**2.73 [2.34, 3.15]**
Infections	725	**1.85 [1.72, 1.99]**	191	**2.05 [1.77, 2.36]**	70	**4.61 [3.59, 5.83]**
Pulmonary	329	1.05 [0.94, 1.17]	35	**1.54 [1.07, 2.14]**	15	**2.76 [1.55, 4.55]**
Renal	135	**1.69 [1.42, 2.00]**	46	**2.09 [1.53, 2.79]**	15	**3.62 [2.03, 5.98]**
Liver and cirrhosis	235	0.90 [0.78, 1.02]	28	1.45 [0.96, 2.09]	14	1.13 [0.62, 1.90]
Complications of pregnancy/childbirth	9	**2.51 [1.15, 4.77]**	5	**4.22 [1.37, 9.85]**	2	5.83 [0.71, 21.08]
Suicide and self-inflicted Injury	362	**1.16 [1.04, 1.29]**	9	1.03 [0.47, 1.96]	9	0.95 [0.44, 1.81]
Accidents and adverse events	663	0.94 [0.87, 1.02]	79	**1.40 [1.11, 1.75]**	58	**2.31 [1.76, 2.99]**
Homicide and legal intervention	55	**0.69 [0.52, 0.90]**	19	0.67 [0.40, 1.05]	6	1.22 [0.45, 2.66]
All other causes	1901	**1.49 [1.42, 1.56]**	293	**1.63 [1.45, 1.83]**	142	**3.02 [2.54, 3.56]**

[1] Includes primary cancer and subsequent malignancy. **Other race = Asian/Pacific Islander/American Indian/Alaska Native. AYA = adolescent and young adult. SMR = standardized mortality ratio; Obs = observed number of deaths. 95%CI = 95% confidence interval.

Table S5. Observed numbers and standardized mortality ratios by cause of death and latency from diagnosis through 30 years, 5-year survivors of all AYA cancers diagnosed 1975–2012, SEER-9

Cause of death	5–10 years from diagnosis		10–20 years from diagnosis		20–30 years from diagnosis	
	**Obs**	**SMR [95%CI]**	**Obs**	**SMR [95%CI]**	**Obs**	**SMR [95%CI]**
All causes of death	9564	**7.41 [7.26, 7.56]**	8448	**2.88 [2.82, 2.94]**	5413	**1.84 [1.80, 1.89]**
Malignant cancers[1]	7798	**27.53 [26.92, 28.14]**	5489	**6.45 [6.28, 6.62]**	2852	**2.83 [2.72, 2.93]**
Cardiovascular/cerebrovascular	378	**1.59 [1.43, 1.75]**	947	**1.40 [1.31, 1.49]**	1023	**1.36 [1.27, 1.44]**
Infections	313	**3.09 [2.75, 3.45]**	347	**1.88 [1.69, 2.09]**	217	**1.56 [1.36, 1.78]**
Pulmonary	18	1.16 [0.69, 1.84]	86	**1.25 [1.00, 1.54]**	155	1.14 [0.97, 1.33]
Renal	18	**1.92 [1.14, 3.03]**	60	**2.18 [1.67, 2.81]**	77	**1.95 [1.54, 2.43]**
Liver and cirrhosis	59	1.24 [0.95, 1.60]	104	0.89 [0.73, 1.08]	78	0.83 [0.65, 1.03]
Complications of pregnancy/childbirth	8	**3.31 [1.43, 6.53]**	8	**3.68 [1.59, 7.25]**	0	0.00 [0.00, 7.89]
Suicide and self-inflicted Injury	120	**1.24 [1.03, 1.49]**	157	1.12 [0.95, 1.31]	76	1.05 [0.83, 1.32]
Accidents and adverse events	237	1.04 [0.91, 1.18]	335	1.03 [0.92, 1.15]	174	1.01 [0.56, 1.66]
Homicide and legal intervention	27	**0.56 [0.37, 0.82]**	36	0.77 [0.54, 1.06]	15	1.01 [0.56, 1.66]
All other causes	448	**2.08 [1.90, 2.29]**	770	**1.59 [1.48, 1.71]**	686	**1.40 [1.30, 1.51]**

[1] Includes primary cancer and subsequent malignancy. AYA = adolescent and young adult. SMR = standardized mortality ratio; Obs = observed number of deaths. 95%CI = 95% confidence interval.

Figure S1. Cause-specific mortality by cause of death and time period conditioned on 5-year survival, all AYA cancers diagnosed 1975–2012, SEER-9

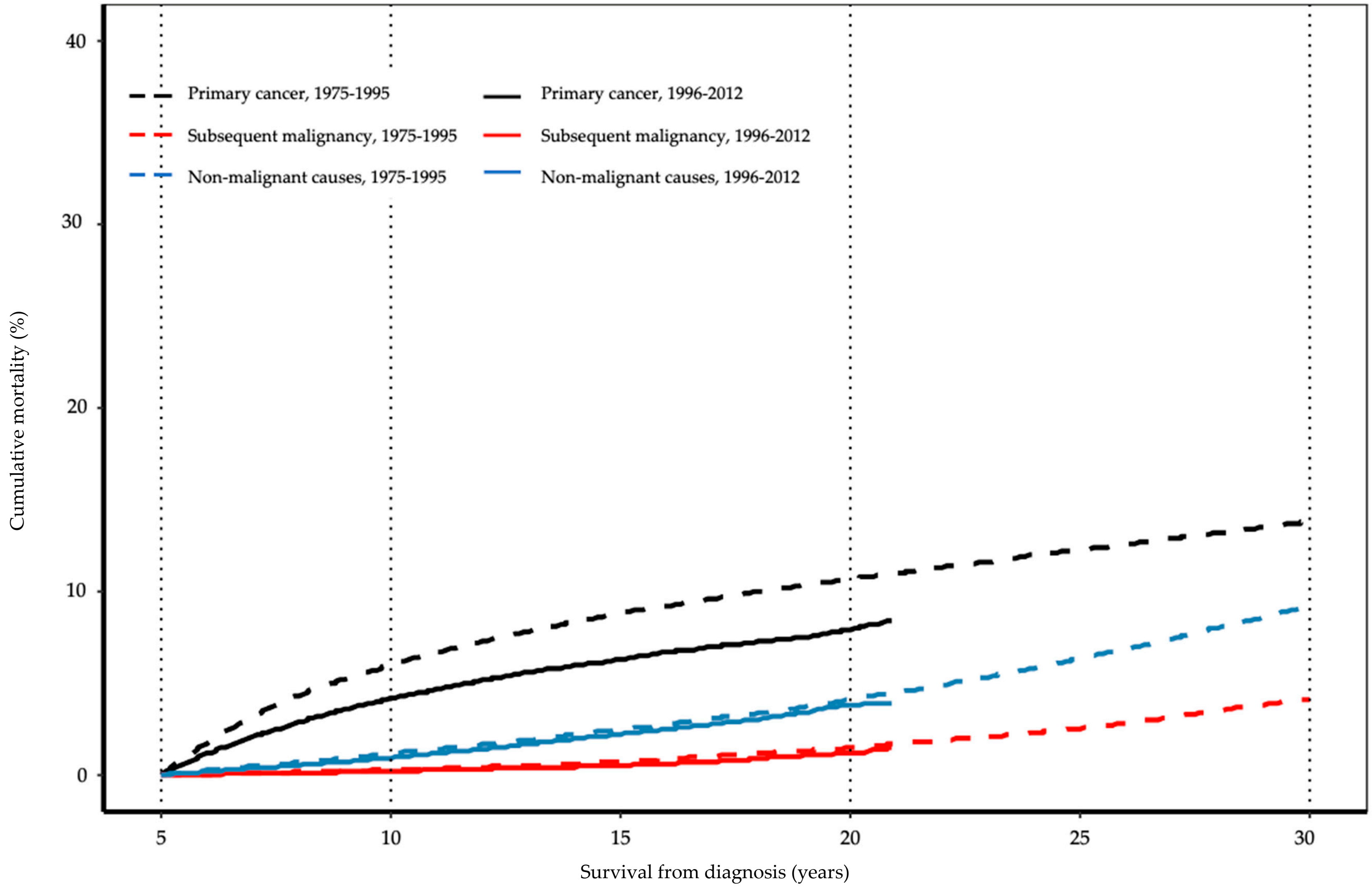

No. at risk

1975–1995	79,771	73,311	69,054	64,599	46,488	27,842
1996–2012	81,436	50,702	25,278	4013	-	-
